# NephroCheck: should we consider urine osmolality?

**DOI:** 10.1186/s13054-019-2341-9

**Published:** 2019-02-14

**Authors:** Alberto Noto, Andrea Cortegiani, Antonio David

**Affiliations:** 1 Department of Anesthesia and Intensive Care, A.O.U. G.Martino, University of Messina, Italy, Via Consolare Valeria 1, 98100 Messina, Italy; 2 Department of Surgical, Oncological and Oral Science (Di.Chir.On.S.), Section of Anesthesia, Analgesia, Intensive Care and Emergency, Policlinico Paolo Giaccone, University of Palermo, Palermo, Italy; 3 National Reaserch Council (CNR), Institute for Chemical and Physical Processes (IPCF), Messina, Italy

Early detection of acute kidney injury (AKI) is challenging due to the risk of morbidity and mortality and a direct impact on patients’ management [1]. The diagnosis relies on the changes of serum creatinine and urine output [2], which are the main markers of kidney function. Recently, Astute Medical introduced the NephroCheck, a test that allows a bedside analysis of two biomarkers of renal damage implicated in G1 cell-cycle arrest: tissue inhibitor metalloproteinase-2 (TIMP-2) and insulin-like growth factor binding protein-7 (IGFBP-7) [3]. The combination of these two biomarkers led to a new score (AKIRisk™). An AKIRisk™ score > 0.3 identifies patients at risk of developing AKI with sensitivity and specificity of 92% and 46%, respectively; increasing the cutoff to 2.0, the sensitivity is 46% and the specificity is 95% [4]. The AKIRisk™ reference interval in healthy humans ranges from 0.04 to 2.22. A possible reason for this wide range could be that the score is not taking into account urine concentration.

We aimed to check the correlation between AKIRisk™ and urine osmolality, using a dehydration test. We collected urine samples from healthy volunteers after 8 h of operating room shift without drinking water (T0) and after drinking 0.5 l of water (T1). Urine samples were analyzed, and osmolality as well as biomarker concentration were measured. Complete measurements are reported in Additional file 1: Table S1. A significant difference was found between the mean AKIRisk™ at T0 (0.82, 95% CI 0.15 to 1.48) vs. T1 (0.24, 95% CI 0.02 to 0.50), *p* = 0.01 (Wilcoxon test—Fig. 1a). The Pearson correlation between osmolality and AKIRisk™ at T0 and T1 was *r* = 0.93, *p* = 0.02, and *r* = 0.80, *p* = 0.03 (Fig. 1b, c).

**Fig. 1:**
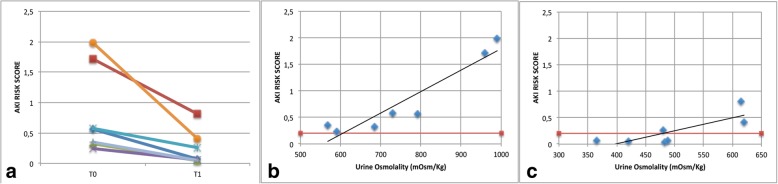
**a** AKIRisk score measured at dehydration (T0) and after hydration (T1). **b**, **c** Relationship between urine osmolality and AKIRisk score measured by NephroCheck at T0 and T1. Red line represents the AKIRisk cutoff

Our results suggest that fluid intake in the normal population is able to modify the urinary concentration of TIMP-2 and IGFB-7. It is to note that every AKIRisk™ > 0.3 occurs in people with urine osmolality > 600 mOsm/kg and that there is a good correlation between urine osmolality and AKIRisk™. Some participants still maintained AKIRisk™ > 0.3 even after fluid reintegration, maintaining a good correlation with urinary osmolality also at T1, indicating a suboptimal dehydration correction.

Our data suggest that the values of AKIRisk™ score could be related to the urine concentration; thus, urine osmolality should be considered in the interpretation of the results of the NephroCheck® test. This correlation should be checked in critically ill patients at risk of AKI.

## Additional file

Additional file 1: Raw data. Urine osmolality and AKIRisk score of each patient. (DOCX 43 kb)
