# Supplementary material for: NephroCheck: should we consider urine osmolality?
Source: Crit Care. 2019 Feb 14;23:48. doi: 10.1186/s13054-019-2341-9 (PMC6376683; doi:10.1186/s13054-019-2341-9)
Supplement: Supplementary file 1 — Raw data. Urine osmolality and AKIRisk score of each patient. (DOCX 43 kb) [file 13054_2019_2341_MOESM1_ESM.docx]

Table 1

| ID | BP | AKIRisk T0 | AKIRisk T1 | Osmolality T0 | Osmolality T1 |
| --- | --- | --- | --- | --- | --- |
| A | 110/70 | 0,56 | 0,07 | 791 | 366 |
| B | 105/70 | 1,72 | 0,81 | 960 | 615 |
| C | 115/75 | 0,32 | 0,04 | 684 | 483 |
| D | 130/90 | 0,24 | 0,06 | 590 | 488 |
| E | 125/70 | 0,58 | 0,26 | 730 | 480 |
| F | 100/60 | 1,98 | 0,41 | 990 | 620 |
| G | 134/72 | 0,35 | 0,05 | 568 | 420 |

BP: blood pressure; AKIRisk score measured at dehydration (T0) and after hydration (T1)
